# Goiter and Laryngopharyngeal Reflux

**DOI:** 10.5402/2012/208958

**Published:** 2012-03-05

**Authors:** Abdul-Latif Hamdan, Jad Jabbour, Zaid Al Zaghal, Sami T. Azar

**Affiliations:** ^1^Department of Otolaryngology and Head & Neck Surgery, American University of Beirut Medical Center, Beirut, Lebanon; ^2^Division of Endocrinology and Metabolism, Department of Internal Medicine, American University of Beirut Medical Center, Beirut, Lebanon

## Abstract

The purpose of this study is to look at the prevalence of laryngopharyngeal reflux disease in patients with goiter in a group of 52 patients with goiter. All participants were asked to respond to the 9 questions on the Reflux Symptom Index (RSI). A diagnosis of LPRD based on symptoms was made for any RSI score above 10. The average score of every question was computed for all patients with goiter and compared to the corresponding average score of the controls. A *P* value of less than 0.05 was considered statistically significant. The total prevalence of LPRD in patients with goiter as indicated by an RSI score greater than 10 was 15.4% versus 9.1% in controls. The difference was not statistically significant (*P* value 0.525). Looking at the average score of the individual symptoms as listed in the RSI questionnaire, the average score of all the symptoms was higher in patients with goiter versus controls. There was no correlation between LPRD and any of the demographic variables except for nodules (*P* value 0.035). The presence of laryngopharyngeal symptoms in patients with goiter should alert the treating physician to the presence of laryngopharyngeal reflux disease.

## 1. Introduction

Goiter is the most prevalent endocrine condition in the world, affecting over 500 million people [1]. Large cross-sectional studies have demonstrated prevalence rates of goiters and/or thyroid nodules between 25 and 33% [2, 3]. In other studies, 26–30% of children [4] have been found to have a goiter long after iodization of salt was standardized in those settings.

Goiters occur as a result of the interplay of genetics and environmental factors, namely, iodine deficiency [5]. The important role of adequate iodine intake and uptake has long been established. Deficient levels lead to hypothyroidism that in turn leads to increased blood levels of TSH, which has a hypertrophic effect on the thyroid [5]. Certain conditions, such as smoking or increased parity, may worsen iodine deficiency and can thus be considered risk factors for goiter [6]. In addition, genetic predisposition plays a crucial role in goiterogenesis [7]. In a region with low iodine support, patients with goiters were found to have significantly higher proportion of parents (*P* < 0.001; OR = 3.6) or siblings (*P* = 0.004; OR = 2.5) with goiters, and patients with a positive goiter family history had a 4.1-fold increased goiter risk. Similarly children of parents with goiters showed a 2.7-fold increased risk for goiter development.

Goiter patients typically present with one or more of a constellation of symptoms that include compressive symptoms such as globus pharyngeus and dysphagia, phonatory symptoms such as hoarseness, cosmetic complaints as an enlarged or palpable neck mass, or airway difficulty due to tracheal compression, shift, or invasion [1]. Many of these symptoms also occur in laryngopharygneal reflux disease (LPRD), which is defined broadly as the backflow of gastric contents into the laryngopharynx. This common condition is found in roughly 50% of patients with a throat-related complaint [8]. The most common symptoms include hoarseness, globus, dysphagia, throat clearing, and cough, whereas the most laryngeal findings are edema, erythema, thick mucus, and muscle tension patterns [9].

Fiorentino et al. [10] examined this very overlap of symptoms by conducting a study on 25 goiter patients who complained of local neck symptoms (throat discomfort, dysphagia, hoarseness). They found that reflux laryngopharyngitis was present in 58–67% of participants, based on video laryngoscopy, and that swallowing alterations, as demonstrated on videofluoroscopic swallowing study, were present in 86–90% [10]. Interestingly, all participants then had a total thyroidectomy as treatment of the goiter; yet, 75% or more continued experiencing the local neck symptoms that had presumably been due to their goiter. The authors concluded these symptoms were likely due to the LPRD, which must also then be addressed in order to relieve patients of the symptoms that originally led them to present. 

No other studies, to our knowledge, have examined the interplay between LPRD and the presence of goiters. Even Fiorentino et al. [10] only approach this question for goiter patients already complaining of local neck symptoms; thus, the select group they study is a biased sample of the overall population of goitrous patients. Thus, the prevalence of LPRD symptoms and findings in all goiter patients (regardless of original complaint) has yet to be investigated. This study is a cross-sectional analysis of the prevalence of reflux symptoms in consecutive patients seen at an endocrinology office for management of goiter. It will also test whether or not there is an association between the two conditions, that is, whether or not patients with goiter have a higher prevalence of LPRD than those without goiter. Improved understanding of LPRD's presence in this patient population will help direct optimal treatment for symptoms that may otherwise be attributed entirely to the presence of a goiter [10].

## 2. Methods

Participants were recruited over a period of two months at a private endocrinology clinic at the American University of Beirut Medical Center. All goiter patients (diagnosed clinically and by ultrasound) between the ages of 18 and 65 were informed of a study taking place in the adjacent Hamdan Voice Unit regarding throat and vocal health and their possible association with goiter. A total of 52 consecutive patients with goiter were recruited for the study. Thirty-three controls matched according to allergy and smoking status were recruited as well. The authors made sure to have the same percentages of smokers and subjects with allergy in both patients and controls in order to address the confounding effect of smoking and allergy in the assessment of laryngopharyngeal symptom. Patients with current upper respiratory infection symptoms or history of laryngeal manipulation were excluded.

Demographic data included age, gender, smoking, allergy using a validated questionnaire [11, 12] duration of disease in years, presence of one or more nodules as documented by ultrasound, and thyroid stimulating hormonal level. A range between 0.27 and 4.20 micro-unit/mL was considered as normal.

All participants were asked to respond to the 9 questions on the Reflux Symptom Index (RSI) [13], and their responses were recorded on the scale by one of two research assistants following an assigned protocol. A diagnosis of LPRD based on symptoms was made for any RSI score above 10. The prevalence of LPRD in patients with hypothyroidism was compared to controls. In addition to the recording of the total score of RSI in all subjects and the frequency of scores above 10 as being indicative of LPRD, the average score of every question was computed for all patients with goiter and compared to the corresponding average score of the controls. A *P* value of less than 0.05 was considered as statistically significant.

Frequencies and means (± standard deviation) were used to describe categorical and continuous variables, respectively. At the bivariate level, normal distribution was not assumed for the total reflux symptoms in Table 3 and *Wilcoxon-Mann and Whitney Rank Sum Test* was used to determine any significant differences in means of each continuous variable when compared between patients and controls and for the duration of disease with RSI. As for the age, the *independent t-test* was used for age in Table 4. *Pearson's chi-square test* was applied for categorical variables in Tables 2 and 4 to assess existence of any correlation. When expected count cells were less than 5, Fisher's exact test was applied instead of Pearson's chi square. All analyses were conducted using the Statistical Package for the Social Sciences version 17 software package. A two-tailed *P* value of less than 0.05 was considered statistically significant.

## 3. Results

### 3.1. Demographic Data

A total of 52 patients with goiter were recruited for this study. The mean age was 47.37 years ± 12.22 years. Eighty-eight per cent were females and almost one-third was smoker. Eleven per cent had history of allergy. The percentage for smoking and allergy was comparable in controls. The mean duration of the disease since diagnosis in patients was 3.32 years ± 4.17. Eight per cent had thyroid gland nodules. With respect to their TSH level, 6 were missing and among those available, 70% were euthyroid at the time of examination. See Table 1.

### 3.2. Prevalence of LPRD and Average Score of LPRD Symptoms in Patients with Goiter and Controls

The total prevalence of LPRD in patients with goiter as indicated by an RSI score greater than 10 was 15.4% versus 9.1% in controls. The difference was not statistically significant (*P* value 0.525). See Table 2.

Looking at the average score of the individual symptoms as listed in the RSI questionnaire, the average score of all the symptoms was higher in patients with goiter versus controls but not significantly higher. See Table 3.

### 3.3. Correlation between LPRD and Goiter Variables

There was no correlation between LPRD and any of the demographic variables except for nodules. Goiter patients with nodules were more likely to have LPRD compared to those with no nodules. See Table 4.

## 4. Discussion

Laryngopharyngeal reflux is among the most common diagnoses made in the laryngologist's office, as roughly 50% of patients with throat or vocal complaints have been shown to have LPRD [8]. The diagnosis is invariably made through a detailed history taking and a laryngeal examination. The presence of many laryngopharyngeal symptoms, such as change in voice quality, throat clearing, globus, and dysphagia, leads to the diagnosis of LPRD. This later is further confirmed by the laryngeal findings of edema, thick mucus, obliteration of the sinus of Morgagni, and the medialization of the false cords among others. The compilation of these findings and complaints under the Reflux Finding score and Reflux Symptom Index by Belafsky are commonly used diagnostic tools that have been validated in comparison to double probe pH-metry. Other diagnostic tests include detection of pepsin in the sputum, laryngeal sensory testing, Laryngopharyngeal sensory discrimination testing (FEESST), esophagoscopy, barium esophagography, and Bernstein acid perfusion [9]. The authors of this paper have elected to use the RSI questionnaire as a diagnostic test for the presence of LPRD in patients with goiter and controls.

Patients with goiter often complain of neck symptoms and disfigurement related to their neck mass. Local symptoms in patients with euthyroid goiter undergoing surgery have been reported to vary between 13 to 50% [14, 15]. Among these are swallowing disorders, voice disturbances, and throat discomfort. These complaints have been linked to the presence of nodules in thyroid diseases and total thyroidectomy has been offered as a treatment option for these patients [15, 16]. The persistence of these neck symptoms following surgery has been linked to the side effects of endotracheal intubation, injury to the perivascular neural plexus innervating the pharyngeal and laryngeal structures, and the normal healing process that is presumed to hinder or impair the vertical movement of the laryngotracheal complex [17, 18].

Recently, Fiorentino et al. [10] suggested that goiter patients may be at risk of undertreatment of LPRD and that many of these symptoms are LPRD symptoms that overlap with those commonly found in cases of goiter. As such the diagnosis of LPRD may be overlooked as all local neck symptoms are attributed to the goiter. Especially in patients whose goiters are only being monitored, and not removed, such symptoms may continue to be endured without any further thought to their etiology. This has been proven by the persistence of swallowing and voice disorders and throat discomfort even three months after surgery. Based on Fiorentino's investigation, these symptoms have been associated with laryngopharyngitis as evidenced by videolaryngoscopy and videofluoroscopic swallowing studies. The results of Fiorentino were in accordance with previous studies by Pereira et al. reporting the persistence of local neck symptoms in 48% of patients undergoing surgery and the emergence of these symptoms subsequent to thyroidectomies in 45% of the cases [14]. 

The results of our study indicate that almost 15.4% of patients with goiter had LPRD. Even though the difference in prevalence was not significant compared to controls, the average scoring grade of all LPRD symptoms was greater in patients with goiter. Several mechanisms can be responsible for the higher prevalence of LPRD and the higher scoring of the above-mentioned symptoms in patients with goiter compared to controls. One is the possible dysfunction of the upper esophageal sphincter (UES) as lower esophageal sphincter pressure in goitrous patients has been previously reported [19]. The UES dysfunction could be attributed to the mass effect of the hypertrophic gland on the cricopharyngeal and constrictor muscles or to vagal neuropathy. The UES is primarily innervated by the recurrent laryngeal nerve (RLN) which can be affected in patients with goiter. In his study on three specimens of the UES obtained from human autopsies examined by Shiler's stain, Mu and Sanders found that the cricopharyngeal (CP) muscle receives its innervation from below via the recurrent laryngeal nerve (RLN) and from above via the pharyngeal plexus and the upper esophagus (UE) is innervated by the RLN [20]. Evidence for RLN dysfunction in patients with benign thyroid lesions exists [21]. Indeed, case reports of both unilateral and bilateral RLN paralysis in patients with benign multinodular goiter have been presented, especially in cases of substernal goiter [22, 23]. The prevalence varies between different studies. Holl-Allen has reported an incidence of 0.69% in 1156 patients with benign thyroid disease, forty percent of which recovered after surgery [24]. His results were further commemorated by many authors reporting similar incidences [25–27]. The basic mechanism behind the paralysis or paresis is perineural inflammation and fibrosis, stretching of the recurrent laryngeal nerve by the hypertrophic gland, compression, and or thrombosis of the vasa nervosa.

 Another explanation for the high prevalence of LPRD in patients with goiter is the delayed esophageal peristalsis reported in these patients by Pustorino [19]. Such peristalsis dysfunction can be further exacerbated if the goiter is accompanied by hypo- or hyperthyroidism [28]. Several studies have reported gastrointestinal motility dysfunction in association with hypo- and hyperthyroidism. Patients with hypothyroidism have been shown to have delayed gastric emptying and disturbed esophageal mobility. A recent study by Yaylali et al. using esophageal scintigraphy on thirty females subjects with hypothyroidism revealed a marked increase in the mean esophageal transit time and gastric emptying, with no correlation between the level of hypothyroidism and abnormalities in gastrointestinal system kinetics [29, 30]. The possible mechanism for these findings is the accumulation of mucinous material (mucopolysaccharides) in the gastrointestinal system mucosa, leading to dysmotility [31, 32]. This possibility is unlikely in our study in view of the fact that 70% of patients with goiter had TSH within normal range.

 The limitation of this study is the lack of laryngeal examination to assess the laryngeal findings secondary to LPRD, and to record the vocal fold mobility in patients with goiter. Nevertheless it is the first study to highlight the prevalence of LPRD in a nonbiased group of patients with goiter.

## 5. Conclusion

Because of the overlap between LPRD symptoms and neck complaints of patients with goiter, it is important to know the prevalence of LPRD in this group of patients. The results of our study indicate a higher prevalence of all laryngopharyngeal reflux disease symptoms in patients with goiter and correlation between LPRD and nodular goiter. As such, goiterous patients need to be treated for LPRD when present, before being scheduled for surgery with the assumption that their neck throat, voice, and swallowing symptoms are secondary to their goiter.

## Figures and Tables

**Table 1:** Descriptive data of patients with goiter and controls.

	Patient (*n* = 52)	Control (*n* = 31)
Age (mean ± SD)	47.37 ± 12.22	39.29 ± 13.32
Gender		
Males	6 (11.5%)	17 (54.8%)
Females	46 (88.5%)	14 (45.2%)
Smoking		
No	33 (63.5%)	20 (64.5%)
Yes	19 (36.5%)	11 (35.5%)
Allergy		
Absent	46 (88.5%)	28 (90.3%)
Present	6 (11.5%)	3 (9.7%)
Duration of disease in years (mean ± SD)	3.32 ± 4.17	N/A
Nodules		N/A
Absent	10 (19.2%)	
Present	42 (80.8%)	
*TSH		N/A
Hyperthyroid	5 (9.6%)	
Euthyroid	36 (69.2%)	
Hypothyroid	5 (9.6%)	
Missing	6 (11.5%)	

*TSH: hyper-thyroid (<0.27); euthyroid (0.27–4.20); hypothyroid (>4.20).

**Table 2:** Reflux among patients and controls.

*Reflux	Patient	Control	*P* value
No	44 (84.6%)	28 (90.3%)	0.525
Yes	8 (15.4%)	3 (9.7%)	

*RSI: No ≤ 10; Yes > 10.

**Table 3:** Reflux symptom index among patients and controls.

	Patients	Controls	*P* value
	mean ± SD	mean ± SD	
Hoarseness or a problem voice	0.87 ± 1.28	0.55 ± 0.85	0.346
Clearing of throat	1.08 ± 1.27	0.77 ± 0.99	0.314
Excess throat mucus or postnasal drip	0.71 ± 1.29	0.42 ± 0.85	0.550
Difficulty swallowing food, liquids, or pills	0.54 ± 1.02	0.26 ± 0.73	0.114
Coughing after you ate or lie down	0.44 ± 0.67	0.35 ± 0.66	0.522
Breathing difficulties or choking episodes	0.31 ± 0.67	0.13 ± 0.43	0.227
Troublesome or annoying cough	0.33 ± 0.83	0.16 ± 0.45	0.532
Sensations of something sticking throat or a lump in throat	0.73 ± 1.07	0.52 ± 0.93	0.280
Heartburn, chest pain, indigestion, or stomach acid coming up	1.35 ± 1.28	1.06 ± 1.37	0.255

**Table 4:** Possible correlations with the total reflux index (RSI).

	Total reflux (RSI)		*P* value
	Absent (≤10)	Present (>10)	
Age (mean ± SD)	47.11 ± 12.21	48.75 ± 13.04	0.731
Duration of disease (mean ± SD)	3.29 ± 4.47	3.50 ± 2.00	0.162
Gender			0.227
Male	4 (66.7%)	2 (33.3%)	
Female	40 (87.0%)	6 (13.0%)	
Nodules			0.035*
Absent	6 (60.0%)	4 (40.0%)	
Present	38 (90.5%)	4 (9.5%)	
TSH			0.103
Hyperthyroid	3 (60.0%)	2 (40.0%)	
Euthyroid	32 (88.9%)	4 (11.1%)	
Hypothyroid	3 (60.0%)	2 (40.0%)	

*Significant results (*P* < 0.05).
